# Time Distributions of Common Respiratory Pathogens Under the Spread of SARS-CoV-2 Among Children in Xiamen, China

**DOI:** 10.3389/fped.2021.584874

**Published:** 2021-04-12

**Authors:** Jinhui Wang, Tiantian Xiao, Feifan Xiao, Shaoxian Hong, Shunqin Wang, Jiancheng Lin, Yong Li, Xiaochuan Wang, Kai Yan, Deyi Zhuang

**Affiliations:** ^1^Department of Clinical Laboratory, Xiamen Children's Hospital (Children's Hospital of Fudan University Xiamen Branch), Xiamen, China; ^2^Department of Neonatology, Children's Hospital of Fudan University, Shanghai, China; ^3^Department of Neonatology, Chengdu Women's and Children's Central Hospital, School of Medicine, University of Electronic Science and Technology of China, Chengdu, China; ^4^Center for Molecular Medicine, Children's Hospital of Fudan University, Institutes of Biomedical Sciences, Fudan University, Shanghai, China; ^5^Pediatric Intensive Care Unit, Xiamen Children's Hospital (Children's Hospital of Fudan University Xiamen Branch), Xiamen, China; ^6^Department of Medical Services, the Second Affiliated Hospital of Xiamen Medical College, Xiamen, China; ^7^Department of Clinical Immunology, Children's Hospital of Fudan University, Shanghai, China; ^8^Xiamen Key Laboratory of Neonatal Diseases, Xiamen Children's Hospital (Children's Hospital of Fudan University Xiamen Branch), Xiamen, China

**Keywords:** children, SARS-CoV-2, viral respiratory infections, influenza, prevention

## Abstract

**Objectives:** The aim of this study was to observe the effect of COVID-19 prevention and control measures on the transmission of common respiratory viruses in a pediatric population.

**Methods:** This was a retrospective observational study. The study population was selected from children with respiratory diseases who attended Xiamen Children's Hospital from January 1, 2018 to January 31, 2021. All children were screened for influenza virus, parainfluenza virus, respiratory syncytial virus (RSV), adenovirus, and Severe acute respiratory syndrome coronavirus 2 (SARS-CoV-2). The changes in respiratory virus detection rates before and after the SARS-CoV-2 intervention were analyzed using an interrupted time-series model. Polynomial curve fitting was also used to predict future short-term trends in respiratory virus detection.

**Results:** A total of 56,859 children were seen at Xiamen Children's Hospital from January 1, 2018 to Jan 31, 2021, of which 32,120 were tested for respiratory viruses via pharyngeal swabs. The overall positive detection rates of the four respiratory viral infections decreased significantly (*P* = 0.0017) after the implementation of the quarantine and school suspension measures in January 2020. Among them, the detection rate of RSV decreased most significantly (*P* = 0.008), and although there was no statistically significant difference in the detection rates of the influenza virus, parainfluenza virus, and adenovirus, a downward trend in the graph was observed. The positive detection rates of RSV in the 0–1-, 1–3-, and 3–7-year-old groups all decreased significantly (*P* = 0.035, 0.016, and 0.038, respectively). The change in the positive detection rate of RSV was relatively stable in the 7–18-year-old group. A total of 10,496 samples were tested for SARS-CoV-2, and no positive cases were reported.

**Conclusions:** The combination of preventive and control measures for COVID-19 reduced the detection rate of four common respiratory viruses, with the greatest impact on RSV. If prevention and control measures continue to be maintained, the overall detection rate or absolute number of detections for the four respiratory viruses will remain low in the short term. However, this trend is likely to vary with the changes in measures.

## Introduction

Viral respiratory infections are common in children and contribute significantly to hospitalization, morbidity, and mortality; thereby increasing the burden on national healthcare budgets (1–3). Therefore, it is important to develop effective prevention strategies. Estimating the annual epidemiological trend of viral respiratory infections would help to guide the development and assessment of prevention efforts and resource planning by health care facilities (4). The year 2020 has been marked by the coronavirus disease 2019 (COVID-19) pandemic. By the end of January 2021, more than 100 million people were infected, and 2,236,284 people had died worldwide (5). Starting from February 2020 (6), China adopted a series of positive measures to deal with the spread of the new coronavirus. Beijing established the National New Coronavirus Prevention Working Group. With provinces as geographical divisions, each province proposed response strategies for prevention and control and adjusted urban emergency levels. Measures included blocking city entrances and strictly strengthening community management and personal protection. Data proved that these measures effectively controlled the further spread of the new coronavirus in a short period of time.

Measures implemented by Xiamen City included closing the city and suspending work and school. On January 23, 2020, the Xiamen Education Bureau notified all universities and primary and secondary schools in Xiamen City to suspend classes and advised parents to allow students to study online and prevent infection by quarantining their homes. As of May 31, 2020, Xiamen schools had not yet reopened. A total of 54 cases of COVID-19 infection were diagnosed in Xiamen, of which 53 were cured and discharged from the hospital. None of the 54 cases of infection was found in children. The implementation of these measures may have also affected the transmission of common respiratory viruses. The aim of this study was to observe the impact of COVID-19 on the transmission of common respiratory viruses in children and to analyze the additional health benefits of integrated protection measures in the prevention and control of respiratory diseases.

## Methods

### Design and Subjects

This retrospective observational study was approved by the ethics committee of Xiamen Children's Hospital. The study included all children with respiratory diseases who were admitted in Xiamen Children's Hospital from January 1, 2018 to January 31, 2021. China began implementing large-scale closures on January 23, 2020. This study was divided into two time periods, before and after the intervention, using January 23 as the time division point.

The inclusion criteria were: (1) acute pharyngitis, acute laryngitis, acute bronchitis, pneumonia, and all other upper and lower respiratory tract infections and lung diseases, such as obstructive pulmonary disease, bronchopulmonary dysplasia, pneumothorax, pulmonary edema, pulmonary abscess, and lung occupancy; and (2) completion of pharyngeal swab screening.

The exclusion criteria were: (1) hospital visits for non-respiratory diseases; (2) repeat respiratory tract infections (one throat swab sampled in Xiamen Children's Hospital within seven days); (3) no otitis media, allergic rhinitis, or sinusitis, with concomitant respiratory tract infections.

### Data Collection

The study collected demographic and admission information, such as the hospitalization ID, gender, age, and hospitalization date, of all children. All data were exported from the Health Information System database.

### Sample Collection

For cases of suspected acute respiratory infection (ARI), throat swab samples for respiratory pathogen detection were routinely collected on the day after admission. When a child was admitted to the hospital, the nurse was responsible for collecting throat swabs. The procedure is as follows: (1) The hospitalization number and name label of the child are checked; (2) The nurse gently wipes the secretions on the palate arch, pharynx, and tonsils on both sides of the child's mouth using a sterile cotton swab; (3) The swab is placed in the culture tube and the lid is closed tightly before transportation.

### Laboratory Testing

Respiratory virus detection: For direct immunofluorescence assay (DFA), swabs specimens were disposed according to the reagent instruction and separated cell spots were stained with fluorescein-conjugated antibody for influenza A and B virus, human parainfluenza virus 1–3 (hPIV1–3), adenovirus (ADV), and respiratory syncytial virus (RSV) (Viral Screening and Identification Kit; Diagnostic Hybrids, USA). The presence of viral antigens in respiratory cells was indicated by the appearance of characteristic intracellular green fluorescence in ≥1 cell. Severe acute respiratory syndrome coronavirus 2 (SARS-CoV-2) real-time reverse transcriptase–polymerase chain reaction tests (Bio-Germ, Shanghai, China) were conducted using throat swab samples.

#### Primary Outcomes

Respiratory virus detection rate is defined as the number of children with a positive throat swab divided by the number of children who received a swab test. A throat swab test result is defined as positive if even one virus (influenza virus, parainfluenza virus, RSV, and adenovirus) is reported.

#### Secondary Outcomes

The influenza virus detection rate is defined as the number of children with a positive throat swab for influenza virus divided by the number of children tested with a throat swab. Parainfluenza virus detection rate is defined as the number of children with a positive throat swab for parainfluenza virus divided by the number of children tested with a throat swab. The RSV detection rate is defined as the number of children with a positive throat swab for RSV divided by the number of children tested with a throat swab. The adenovirus virus detection rate is defined as the number of children with a positive throat swab for adenovirus virus divided by the number of children tested with a throat swab.

### Sample Size Calculation

The study reviewed all children admitted to Xiamen Children's Hospital from January 1, 2018 to January 31, 2021, and investigated the entire group of hospitalized children during this period.

### Quality Control

After collecting demographics and other data, the investigators checked the outliers and missing numbers, and contacted the family members of the children by telephone to make corrections. Throat specimens were collected using the same operating method mentioned above, and all samples were tested by the inspection department of Xiamen Children's Hospital.

### Statistical Analysis

The study used Microsoft Office Excel 2016 to build the database, and Stata 15.0 statistical software was used to complete the statistical analysis. All count data were described in terms of frequency, and all measurement data were described in terms of mean and standard deviation. The Chi-square test was used to compare the rate between two independent groups. With considerations to the possible changes in respiratory virus detection rates as influenced by quarantine and school closure measures and seasonal factors, we used the interrupted time series analysis to compare differences in the detection rates of the four respiratory viruses before and after quarantine and school closure measures. In addition, we applied polynomial curve fitting to predict the absolute number of respiratory virus detections and detection rates in the short term. The inspection level was set to 0.05. When the *P*-value was < 0.05, it was considered statistically significant.

## Results

### General Description

From January 1, 2018 to December 31, 2020, a total of 55,491 children aged 0–18 years, were hospitalized in Xiamen Children's Hospital. The number of hospitalized children in Xiamen Children's Hospital accounts for nearly 1/5 of the total number of hospitalized children in Xiamen (Supplementary Figure 1).

From January 1, 2018 to January 31, 2021, a total of 32,120 children (19,203 [59.79%] males and 12,917 [40.21%] females) were tested for respiratory viruses. This accounted for 55.39% of the total number of hospitalized children in Xiamen Children's Hospital. The median age and range were 2.3 (0, 17.5), including 5,269 (16.4%) in the 0–1-year-old group; 14,560 (45.33%) in the 1–3-year-old group; 10,125 (31.52%) in the 3–7-year-old group, 2,079 (6.47%) in the 7–12-year-old group; and 87 (0.27%) in the 12–18-year-old group (Table 1). In the past years (January 1, 2018 to January 31, 2021), a total of 5,824 (18.13%) pediatric cases of the four common respiratory viral infections were detected, including 760 (2.37%) cases of influenza virus infections, 1,052 (3.28%) cases of parainfluenza virus infections, 3,144 (9.79%) cases of RSV infections, and 868 (2.70%) cases of adenovirus infections. After the implementation of the isolation measures, we conducted a SARS-CoV-2 nucleic acid test on all the hospitalized children. A total of 10,496 samples were tested during this period, and all were negative (100%).

**Table 1:** Baseline for children admitted to Xiamen Children's Hospital and tested for respiratory pathogens from January 1, 2018 to January 31, 2021.

		**2018**		**2019**		**2020**		**2021**	
		***n***	**%**	***n***	**%**	***n***	**%**	***n***	**%**
**Gender**	Male	5,546	61.7	7,417	59.68	5,468	58.57	772	56.46
	Female	3,442	38.3	5,011	40.32	3,868	41.43	596	43.54
**Age (years)**	0–1	1,675	18.64	1,686	13.57	1,700	18.21	208	15.18
	1–3	4,377	48.7	5,380	43.29	4,239	45.4	564	41.24
	3–7	2,481	27.6	4,300	34.6	2,943	31.52	401	29.32
	7–12	428	4.76	1,021	8.22	446	4.78	184	13.43
	12–18	27	0.3	41	0.33	8	0.09	11	0.83
**Flu**	Negative	8,722	97.04	12,226	98.37	9,079	97.25	1,333	97.44
	Positive	266	2.96	202	1.63	257	2.75	35	2.56
**PIV**	Negative	8,624	95.95	11,989	96.47	9,119	97.68	1,336	97.66
	Positive	364	4.05	439	3.53	217	2.32	32	2.34
**RSV**	Negative	7,639	84.99	11,338	91.23	8,652	92.67	1,347	98.46
	Positive	1,349	15.01	1,090	8.77	684	7.33	21	1.54
**ADV**	Negative	8,773	97.61	11,823	95.13	9,289	99.5	1,367	99.93
	Positive	215	2.39	605	4.87	47	0.5	1	0.07
**SARS-CoV-2**	Negative	/	/	/	/	9,128	100	1,368	100
	Positive	/	/	/	/	0	0	0	0

Children aged 1–3 years accounted for nearly half of all positive tests (2,885, 49.54%). The number of positive results in the two age groups of 0–1 and 3–7 years were similar (1,386, 23.8%, 1,280, 21.98%). The number of positive results in the age group of 7 years and above was relatively small.

### Comparison of the Number of Positive Detections in Different Age Groups Over a 3-Year Period

**Figure 1:**
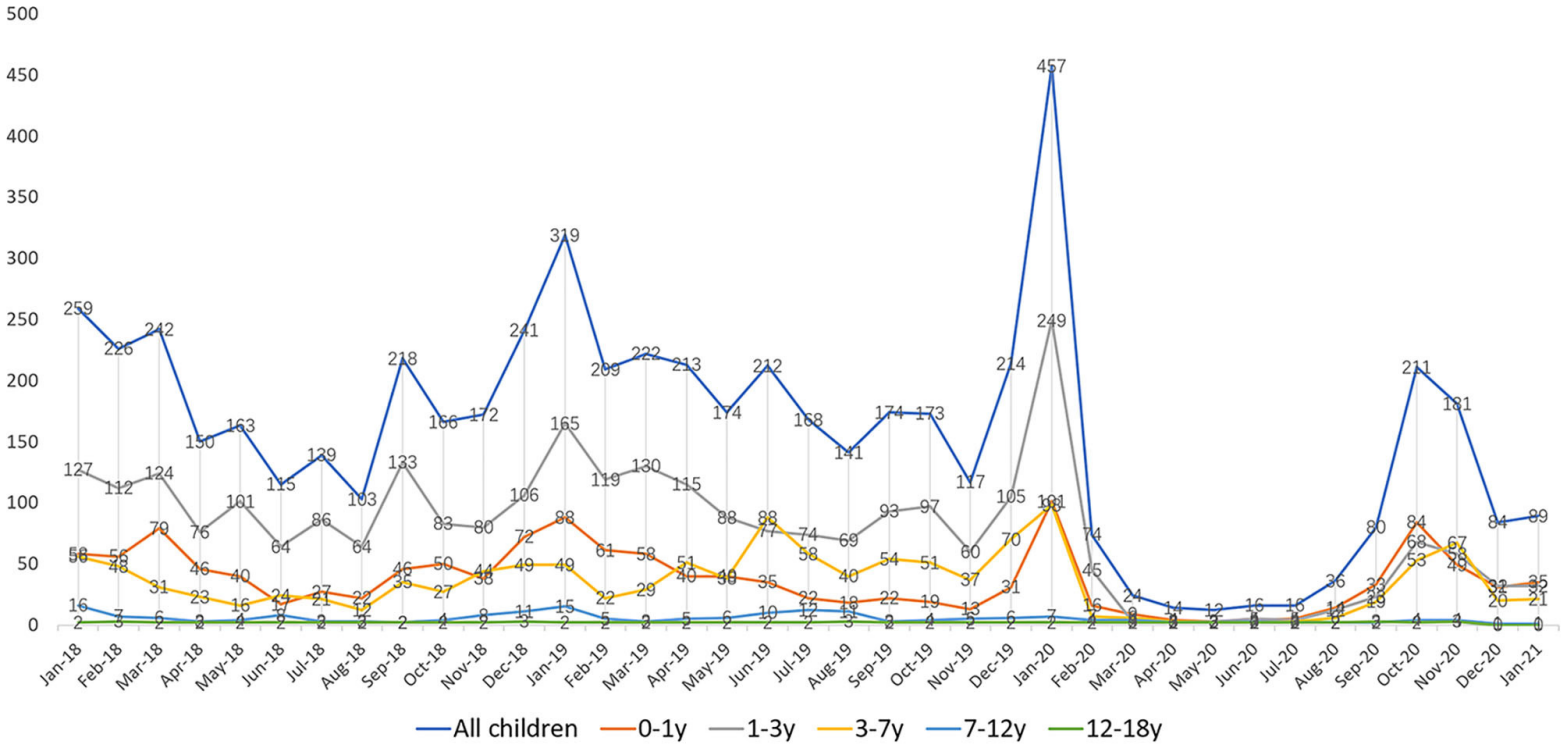
Infection of four major respiratory viruses in children of different ages from January 1, 2018 to January 31, 2021.

Figure 1 presents an interesting phenomenon. Over the 2-year period from January 2018 to January 2020, children aged 1–3 years had the highest number of positive detections for the four common respiratory viruses (2,348, 51.83%). The number of positive detections was similar for the age groups 0–1 and 3–7 years (998, 22.03%; 973, 21.48%), with a lower number of positive detections in the age group 7 years and older. From January 2020 after the outbreak, the percentage of positive children aged 1–3 years (537, 41.50%) showed a decrease, but the percentage was still higher than in the 0–1 (388, 29.98%) and 3–7 years age groups (307, 23.72%) (*P* < 0.001). However, the number of positive detections was similar in the three age groups in terms of monthly trends.

### Comparison of the Number of Positive Detections Between Different Pathogens Over a 3-Year Period

**Figure 2:**
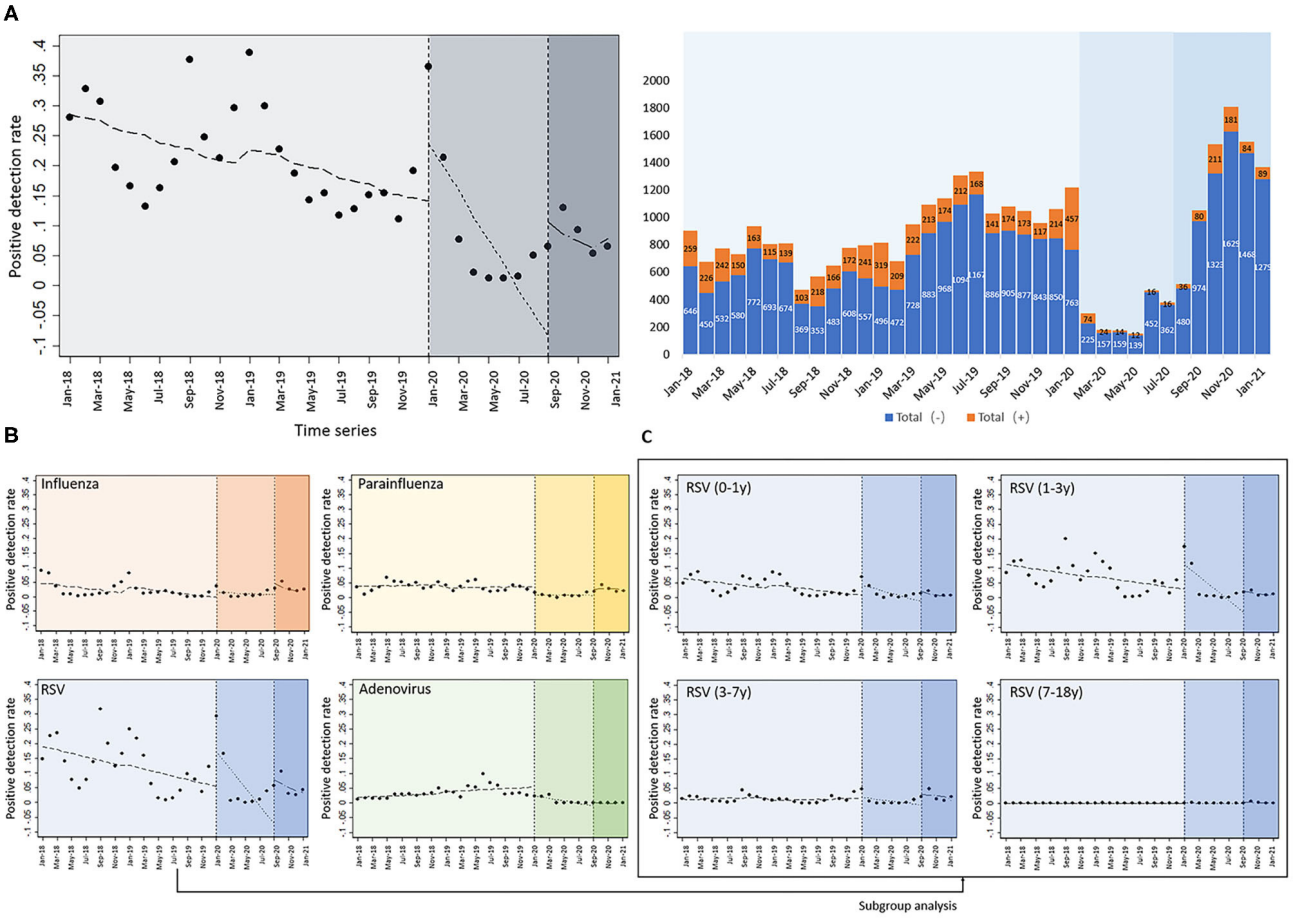
Interrupted time series analysis of positive detection rates of four common respiratory viruses. **(A)** The absolute number of detections and detection rates of the four respiratory viruses after the implementation of isolation and school closure measures, after adjusting for seasonal factors; **(B)** detection rates of influenza virus, parainfluenza virus, and adenovirus; **(C)** subgroup analysis of RSV positive detection rates according to the different age groups.

Using an interrupted time series model, we divided January 2018–2021 into three periods. The first period was before the SARS-COV2 outbreak (January 2018 to December 2019), the second period was the full quarantine and work and school suspension measures in China after the outbreak (January 2020 to August 2020), and the third period was the return to work and school after the SARS-COV2 outbreak was controlled (September 2020 to January 2021). We compared the positive detection rates for the four respiratory viruses in the three periods adjusted for seasonal factors (Figure 2A). We found a significant decrease in the overall positive detection rate for the four respiratory viral infections after the implementation of quarantine and school closure in January 2020 (*P* = 0.0017). The most significant decrease in detection rate was observed for RSV (*P* = 0.008). Although there was no statistically significant difference in the detection rates of influenza virus, parainfluenza virus, and adenovirus, we can still see a flatter decreasing trend in the graph (Figure 2B). Positive detection rates for the four viruses increased after the resumption of work and school in September 2020 and then decreased again, and no rebound trend in positive detection rates due to the resumption of work and school was seen. We further performed subgroup analysis of RSV positive detection rates according to different age groups (Figure 2C) and found that positive detection rates in the 0–1, 1–3, and 3–7-year-old groups decreased significantly after quarantine and school suspension measures (*P* = 0.035, 0.016, and 0.038, respectively). In addition, the change in the positive detection rate was relatively stable in the 7–18 years group.

Supplementary Figure 2 shows the temporal distribution of the absolute number of positive detections of the four common respiratory viruses in Xiamen Children's Hospital. In terms of the absolute number of positive detections of the four common respiratory viruses among inpatients, there is a small peak in September and October each year. The highest number of positive detections still occurred during the winter months (January each year from 2018 to 2020, but not the usual peak in 2021), especially for influenza and RSV viruses. The peak of positive detections for RSV viruses was in September each year and in the spring of the following year. there was still one small peak for RSV after the resumption of school measures in September 2020; however, the number of positive RSV detections in the winter was significantly lower than in the previous 2 years, and the previous peak that might have occurred in January did not occur. The small peak in positive adenovirus detections occurred in the summer from 2018 to 2019, and did not occur in the summer of 2020. A small peak in positive detections with parainfluenza virus occurred in the spring and summer (around May) from 2018 to 2019, but did not occur in the spring and summer of 2020. There was still a small peak in the number of positive detections with parainfluenza virus after the school year resumed in September, 2020, but there was a rapid decline in November. Influenza did not show an epidemic trend for the whole year of 2020, showing a very low number of positive detections. The absolute number of children positively detected for common respiratory viruses reached its lowest level in 3 years from March to July 2020.

### Prediction of Absolute Number of Positive Detections and Positive Rate

**Figure 3:**
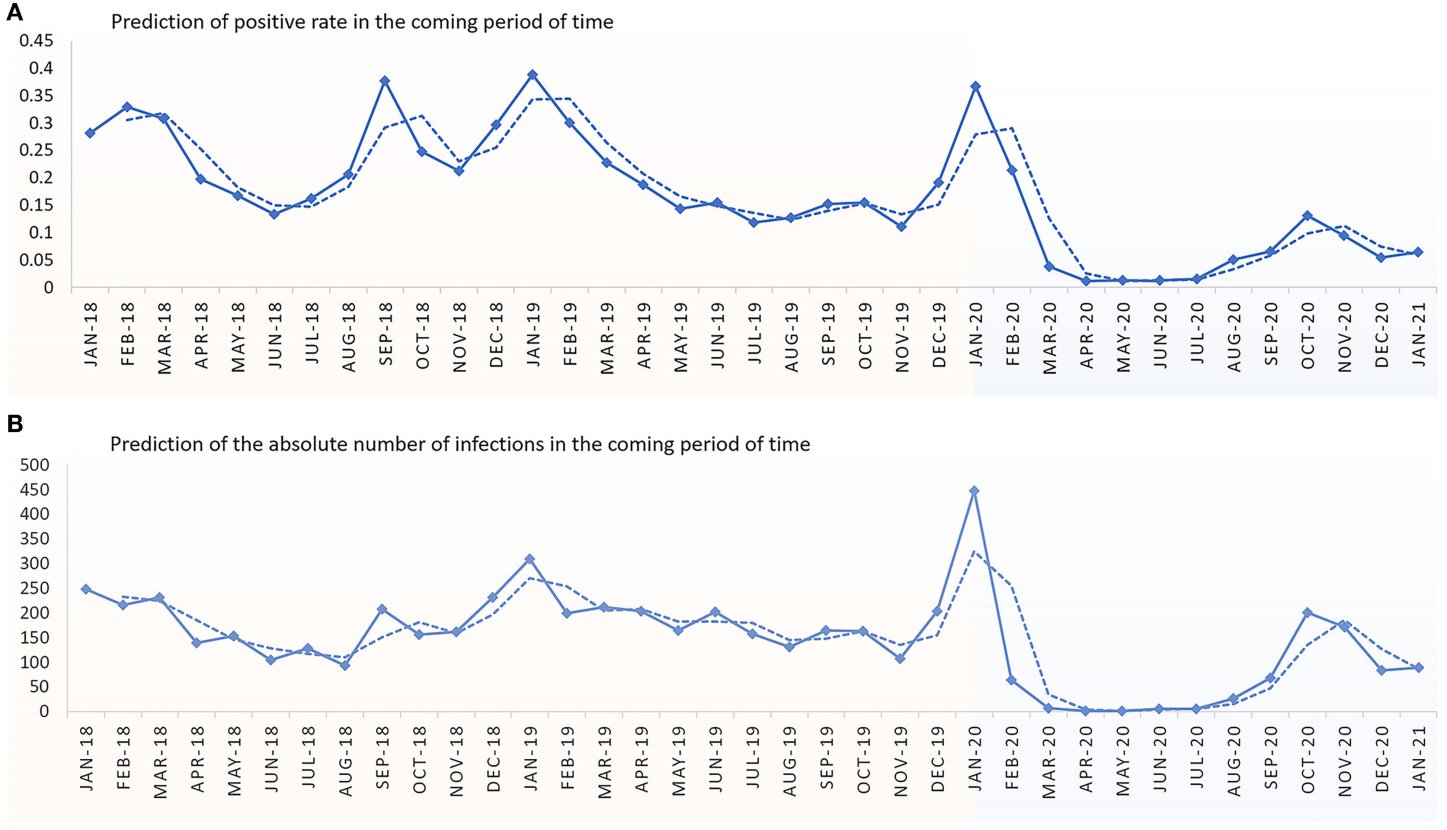
Prediction of the absolute number of infections and the positive rate of children hospitalized in Xiamen Children's Hospital in the next months of 2021. **(A)** Prediction of positive rate in the coming period of time. **(B)** Prediction of absolute number of infections in the coming period of time.

We used a polynomial curve fitting based on the available infections to fit the prediction function from the training and observation sets so that it was as close as possible to the absolute number of actual detections and the detection rates. The fitting results show that the overall positive rate (Figure 3A) or absolute number of infections (Figure 3B) of the four respiratory viruses will remain at a low level in the next months of 2021. However, this trend may be affected by changes in national and local government measures to prevent epidemics.

## Discussion

Common ARI viruses included RSV, influenza, parainfluenza, adenovirus, human metapneumoviruses, and picornaviruses, with epidemics occurring every winter (7–9). Consistent with the findings from other studies (7–10), our study results showed that major viral pathogens causing ARI in children included RSV (9.79%), influenza virus (2.37%), parainfluenza virus (3.28%), and adenovirus (2.70%) in different seasons. As expected, children under 3 years of age were more susceptible to respiratory viruses. In addition, the positive detection rate of RSV was higher than that of influenza virus, parainfluenza virus, and adenovirus among children <3 years of age. A previous report indicated that common respiratory viruses were detected throughout the year, with a peak in winter (11). However, in December 2019, an outbreak of COVID-19 caused by SARS-CoV-2 occurred in Wuhan, China, and it spread rapidly worldwide. Our study showed that the nationwide public health interventions since January 23, 2020, including personal hygiene and protection, face masks, social distancing, home confinement, traffic restriction, centralized quarantine, and effective international surveillance, not only mitigated the spread of SARS-CoV-2 outside of Wuhan, but also effectively decreased the numbers of children with common viral respiratory infections since February 2020. As the epidemic continued until March–May 2020, the implementation of public health interventions did not stop. After the resumption of work and school in September 2020, there was a brief increase in the number of positive respiratory virus tests (limited to September), which may be related to close contact between children indoors after the resumption of school. The subsequent rapid decline in positive detections may be related to local government measures targeting high-risk groups requiring completion of influenza vaccination, as well as requiring students to wear masks and improve hand hygiene in the classroom. All these measures may have been effective in reducing the positive detection rate. Accordingly, our study predicted that the number of children with positive detection of the four common viruses may continually decrease in the next months of 2021.

Interestingly, in our study, the positive detection rate of RSV was significantly lower than that during the same period in 2019. Several lines of evidence have supported that RSV infection is associated with acute lower respiratory tract infection and asthma, leading to an increased risk of mortality and morbidity in children (12–14). In addition, RSV infection in children can place a burden on health services each winter (15). Therefore, a study has evaluated the effectiveness of various measures to decrease the positive rate of RSV (16). Our study indicated that the positive rate of RSV could be decreased with strict quarantine, face masks, and limitation of interactive and school activities. In addition, our study findings showed that the total number of children with influenza virus, parainfluenza virus, and adenovirus infections decreased during the pandemic period, but their positive detection rates were like those during the same period in 2019, even with the strict public health interventions. Although the transmission of these respiratory viruses may occur via aerosol, large respiratory droplets, or contact with contaminated fomites, the mode of transmission for each may be different. Therefore, prevention and control strategies may be adjusted according to the type of virus. Our study suggests that nationwide public health interventions may not be effective for all respiratory viruses, such as adenovirus and parainfluenza virus, and that appropriate approaches may need to be selected for specific respiratory pathogens to enhance prevention.

In our study, no children with SARS-CoV-2 infection were identified in Xiamen after the metropolitan-wide quarantine in Wuhan, which may indicate the low incidence of the SARS-CoV-2 infection in children outside of Wuhan. Therefore, medical costs for national SARS-CoV-2 screening in children may be not necessary. Instead, targeting the at-risk groups for COVID-19 may facilitate a cost-efficient strategy for the national prevention program.

To our knowledge, the interrupted time series (ITS) is one of the strongest quasi-experimental designs. Our study was performed in the pandemic period, and some potential biases in our observational study design were difficult to avoid. Studies have indicated that ITS analysis on evaluation of healthcare interventions was increasingly advocated because of high-quality control for the secular trends in healthcare outcomes (17). Therefore, we performed the ITS to analyze our data.

Our study is the first to delineate the epidemiology of common respiratory viral infections among children aged 0–18 years in Xiamen after the metropolitan-wide quarantine in Wuhan. But there are several limitations that should be addressed. First, our data came from a single hospital in Xiamen; hence, generalizability may be limited (Supplementary Figure 1). Second, we only presented data in a short time period after implementation of COVID hygiene measures. Although we predicted the number of infections and positive rate in the remaining months of 2020, these may be influenced by changes in home isolation and school closure measures. A growing number of scholars believe that SARS-COV2 will coexist with humans for a long time. This means that we need to continue to find effective measures to contain the outbreak to the best of our ability. Although Figure 3 shows that the number of positive detections may remain at a low level, these may be influenced by changes in home isolation and school closure measures. We can also see from Figure 2 that the return to work and school has a significant impact on the rate of positive detections. Notably, some measures (such as wear face mask) still exist in Xiamen. Thus, study about the impact of these measures will be done in the future. Third, there are many public health interventions implemented during the pandemic period, and we did not further discuss which of these strategies may be the most effective in preventing respiratory viral transmission. Fourth, although we adjusted our data based on seasonal factors, the influences of seasons (such as temperature, humidity, and the mood of people) still exist. Finally, regarding the viral detection methods, DFA via throat swabs is the routine method performed in our hospital, but it has low sensitivity.

In conclusion, the study demonstrated that with the metropolitan-wide quarantine in Wuhan and effective national public health interventions, the incidence of other respiratory viral infections considerably decreased among children aged 0–7 years, and SARS-CoV-2 was not detected among pediatric patients in Xiamen. In the remaining months of 2021, owing to the continued public health interventions, the low positive rate of respiratory viral infections is likely to continue, and the positive detections rate of RSV among children <3 years of age may also decrease. These findings may help to improve public health responses and guide the introduction of cost-effective interventions in the next months of 2021. Considering the mentioned limitations, a follow-up study about the RSV positive rate will be performed.

## Data Availability Statement

The raw data supporting the conclusions of this article will be made available by the authors, without undue reservation.

## Ethics Statement

The studies involving human participants were reviewed and approved by the ethics committee of Xiamen Children's Hospital. Written informed consent to participate in this study was provided by the participants' legal guardian/next of kin.

## Author Contributions

JW, TX, KY, and DZ contributed to the conception and design of the study. FX, SH, SW, JL, YL, TX, and XW collected and assembled data. FX, SH, SW, JL, YL, TX, and XW performed the statistical analysis. All authors contributed to manuscript revision, read, and approved the submitted version.

## Conflict of Interest

The authors declare that the research was conducted in the absence of any commercial or financial relationships that could be construed as a potential conflict of interest.

## Supplementary Material

Supplementary Figure 1 Overview of the number of hospitalized children in Xiamen from January 1, 2018 to December 31, 2020. In each line, the orange part plus the gray part is equal to the number of children hospitalized in Xiamen Children's Hospital; the orange part plus the gray part plus the blue part is equal to the total number of children hospitalized in Xiamen.

Supplementary Figure 2 Time distribution characteristics of different viral infections in hospitalized children from January 1, 2018 to January 31, 2020.
