# Supplementary figures and images for: Time Distributions of Common Respiratory Pathogens Under the Spread of SARS-CoV-2 Among Children in Xiamen, China
Source: Front Pediatr. 2021 Apr 12;9:584874. doi: 10.3389/fped.2021.584874 (PMC8075055; doi:10.3389/fped.2021.584874)

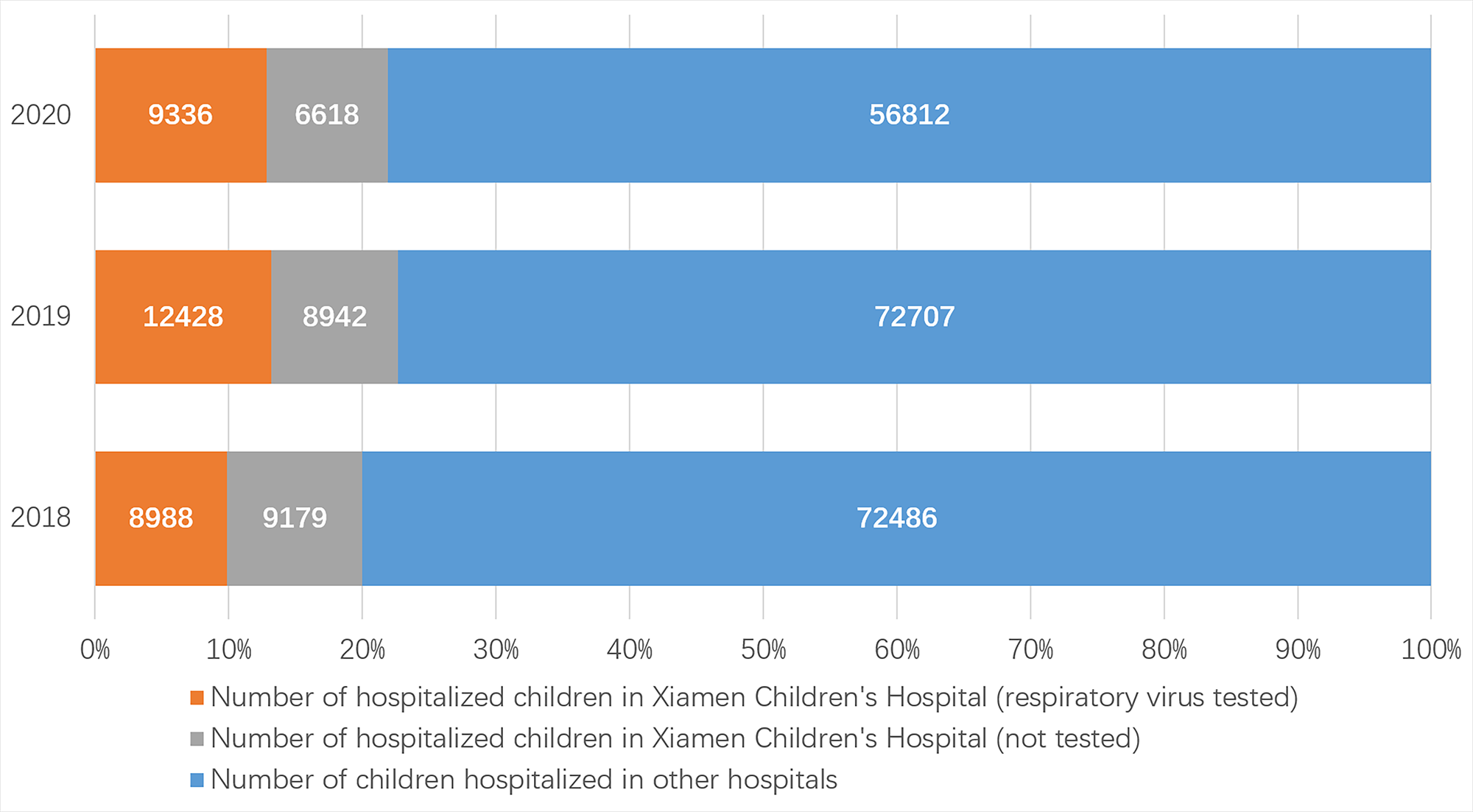

Supplement: Supplementary Figure 1 — Overview of the number of hospitalized children in Xiamen from January 1, 2018 to December 31, 2020. In each line, the orange part plus the gray part is equal to the number of children hospitalized in Xiamen Children's Hospital; the orange part plus the gray part plus the blue part is equal to the total number of children hospitalized in Xiamen. [file Image_1.TIF]

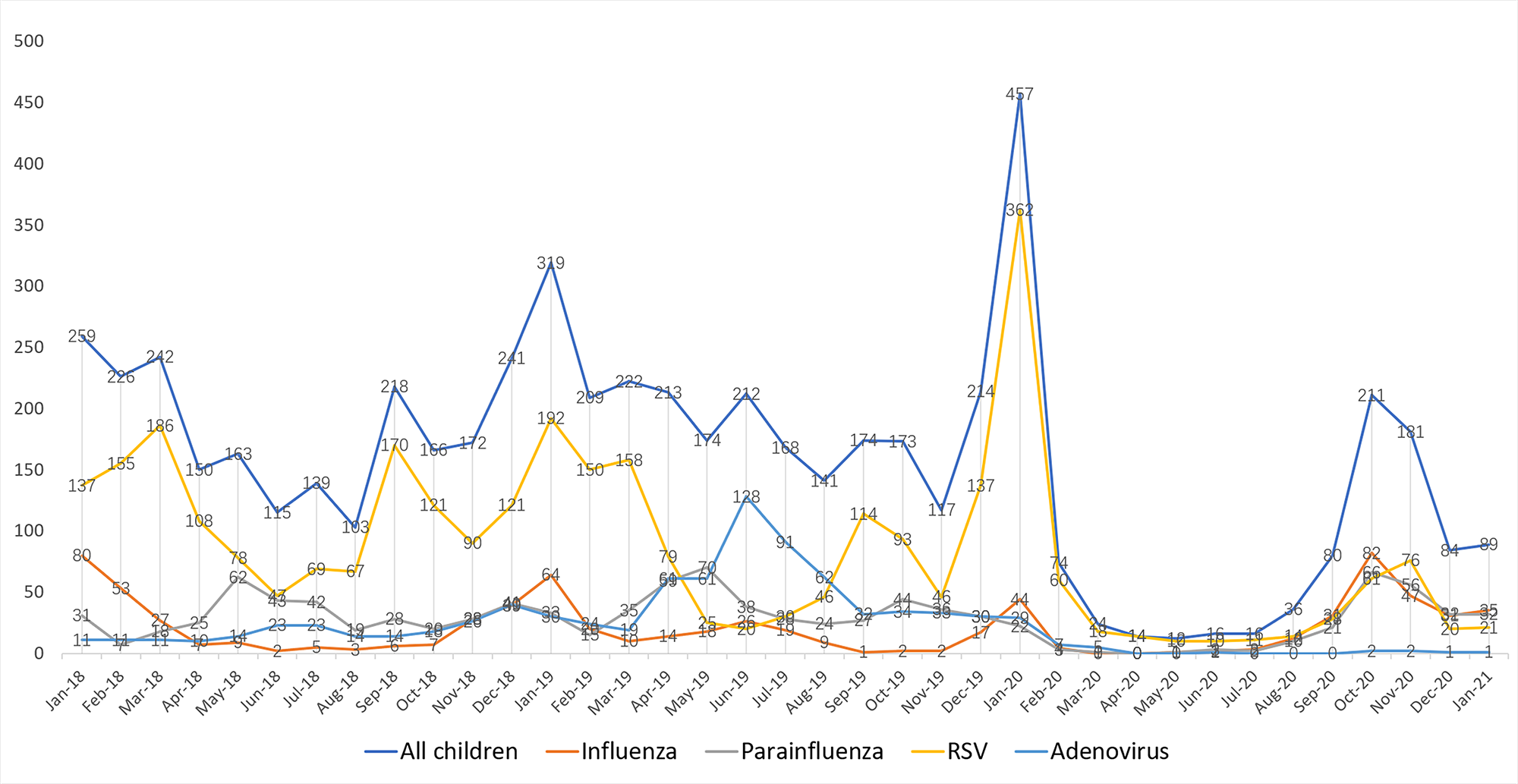

Supplement: Supplementary Figure 2 — Time distribution characteristics of different viral infections in hospitalized children from January 1, 2018 to January 31, 2020. [file Image_2.TIF]
